# Effect of canine cortical bone demineralization on osteogenic differentiation of adipose-derived mesenchymal stromal cells

**DOI:** 10.1016/j.heliyon.2017.e00383

**Published:** 2017-08-17

**Authors:** Kwangrae Jo, Yongsun Kim, Seung Hoon Lee, Yong Seok Yoon, Wan Hee Kim, Oh-Kyeong Kweon

**Affiliations:** BK21 PLUS Program for Creative Veterinary Science Research, Research Institute for Veterinary Science, College of Veterinary Medicine, Seoul National University, Gwanak-ro 1, Seoul 08826, Republic of Korea

**Keywords:** Physiology, Stem cells research, Anatomy, Nanotechnology, Materials science, Biomedical engineering, Surgery, Pharmaceutical science, Bioengineering, Biotechnology

## Abstract

Demineralized bone allografts and mesenchymal stromal cells have been used to promote bone regeneration. However, the degree to which cortical bone should be demineralized for use in combination with adipose-derived mesenchymal stromal cells (Ad-MSCs) remains to be clarified. In this study, the *in vitro* osteogenic ability of Ad-MSCs on allografts was investigated in relation to the extent of demineralization. Three treatment groups were established by varying exposure time to 0.6 N HCL: partially demineralized (PDB; 12 h), fully demineralized (FDB; 48 h), and non-demineralized bone (NDB; 0 h, as a control). Allografts were prepared as discs 6 mm in diameter for *in vitro* evaluation, and their demineralization and structure were evaluated by micro-computed tomography and scanning electron microscopy. Ad-MSC adhesion and proliferation were measured by MTS assay, and osteogenesis-related gene expression was assessed by quantitative reverse transcription polymerase chain reaction. PDB and FDB demineralization rates were 57.13 and 92.30%, respectively. Moreover, Ad-MSC adhesion rates on NDB, PDB, and FDB were 53.41, 60.65, and 61.32%, respectively. Proliferation of these cells on FDB increased significantly after 2 days of culture compared to the other groups (*P* < 0.05). Furthermore, expression of the osteogenic genes *ALP*, *BMP-7*, and *TGF-β* in the FDB group on culture day 3 was significantly elevated in comparison to the other treatments. Given its biocompatibility and promotion of the osteogenic differentiation of Ad-MSCs, our results suggest that FDB may be a suitable scaffold for use in the repair of bone defects.

## 1 Introduction

Bone allografts have been successfully used as an alternative tissue engineering material for bone regeneration [1, 2]. Cortical bone contains bone morphogenetic proteins (BMPs) and matrix proteins. These former are osteoinductive glycoproteins that participate in bone formation [3] and can be activated by demineralization of cortical bone. Osteoclasts or macrophages degrade an allograft at the implantation site, digesting the calcium component, before blood vessels and osteoblasts move into the demineralized construct, resulting in new bone formation [4].

Mesenchymal stromal cells (MSCs) are an attractive therapeutic resource for bone tissue repair [5]. MSCs derived from adipose and placental tissue, bone marrow, muscle, and umbilical cord blood are capable of osteoblastic lineage differentiation under osteogenic culture conditions [6]. Bone formation using adipose-derived MSCs (Ad-MSCs) requires suitable scaffolds for the adhesion and proliferation of these cells. Type I collagen is the major organic component of the extracellular matrix of bone and interacts with MSCs, contributing to the bone healing process [7]. Demineralization by hydrochloric acid exposes type I collagen fibers in bone through the removal of mineral components. Certain synthetic scaffolds, such as those composed of tricalcium phosphate, hydroxyapatite, and poly (methyl methacrylate), exhibit a high level of mechanical stiffness but achieve relatively poor cell seeding efficiency and inadequate cell distribution [7]. Demineralized bone allografts not only consist of a natural biomaterial, but are also osteoinductive and accelerate bone regeneration by eliminating the time needed to decalcify non-demineralized bone allografts *in vivo*.

Demineralized cortical bone may be used as a suitable scaffold for therapy in combination with Ad-MSCs. However, the optimal degree of demineralization to promote osteogenic differentiation of these cells has not been determined. The present study was designed to evaluate the *in vitro* biocompatibility and osteogenic potential of canine Ad-MSCs according to the extent of bone allograft demineralization.

## 2 Materials and methods

### 2.1 Bone harvesting and demineralization

**Fig. 1:**
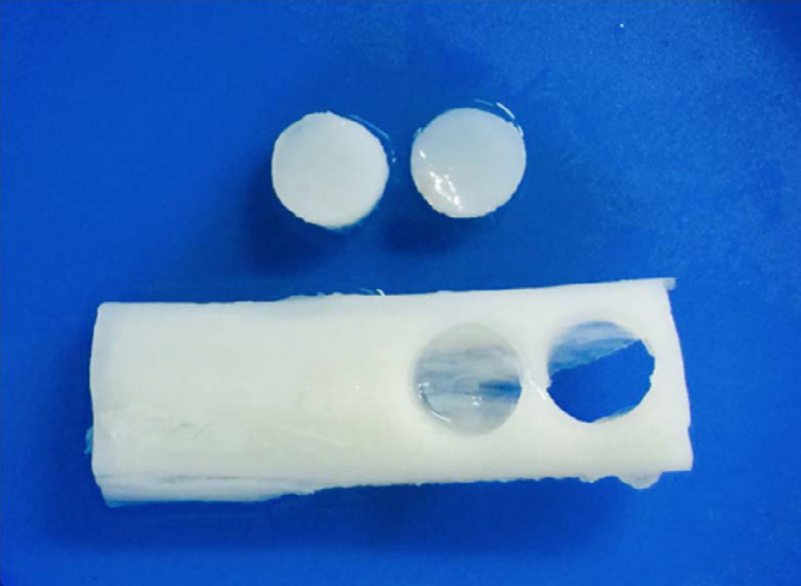
Shape of bone discs. Bone discs were 6 mm in diameter and collected by punch biopsy.

Cortical bone samples were harvested from adult beagles. All animal experimental procedures were approved by the Institutional Animal Care and Use Committee of Seoul National University (SNU-160831-6). Demineralization of these specimens was performed as previously described [8, 9], with modifications. Briefly, cortical bone samples were cut with an oscillating saw into sections approximately 2 cm in length and 1 cm in diameter. These were then soaked in absolute ethanol for 8 h. Decellularization was achieved by exposure to 1% Triton X–100 for 48 h. Degreasing was performed in anhydrous ethyl ether for 12 h, before demineralizing samples in the partially (PDB) and fully demineralized bone (FDB) groups in 0.6 N HCl (50 ml/g of bone) for 12 and 48 h, respectively, followed by soaking in sterile phosphate buffered saline (PBS) for 24 h to neutralize the acidic pH. Non-demineralized bone (NDB) control samples were not treated with HCl. The cortical bone specimens were subsequently dried at room temperature for 24 h and used to make bone discs with a 6 mm biopsy punch (Miltex GmbH, Rietheim-Weilheim, Germany; Fig. 1). The latter procedure was carried out in sterilized distilled water to minimize heat damage.

### 2.2 Isolation and culture of Ad-MSCs

Canine Ad-MSCs were obtained as described in our previous report [10]. Adipose tissues were collected from dogs under general anesthesia from the gluteal region. The collected tissues were repeatedly washed with Dulbecco’s PBS (DPBS; Gibco, Grand Island, NY, USA) and minced using scissors, before being digested with collagenase type I (1 mg/ml; Sigma-Aldrich, St. Louis, MO, USA) at 37 °C for 2 h. Following further washing in DPBS, the tissues were centrifuged at 980 × *g* for 10 min at 4 °C, and the resulting cell pellet was resuspended in low-glucose Dulbecco’s modified Eagle’s medium (DMEM; HyClone, Logan, UT, USA) containing 10% fetal bovine serum (FBS; Gibco), 1 U/mL penicillin(HyClone) and 1 μg/mL streptomycin (HyClone). Cells were then cultured at 37 °C in 5% CO_2_, with the medium being changed every 2 days to remove unattached cells. When confluency reached 90%, cells were subcultured. Third passage cells were used in subsequent experiments.

### 2.3 Cell seeding

Suspensions of 2 × 10^4^ Ad-MSCs in 200 μl low-glucose DMEM containing 10% FBS, 1 U/mL penicillin and 1 μg/mL streptomycin were seeded onto each bone disc in 96-well plates, which were then incubated at 37 °C in 5% CO_2_ for comparison of adhesion and proliferation rates among groups. The cells were left on the disk for 24 h to give them enough time to attach because of the different disc surface characteristics. Cultures were maintained for up to 3 days under these conditions_,_ with the medium being replaced every 24 h. Cell-free bone discs and cell-seeded scaffolds cultured in growth medium were processed alongside the samples as controls.

### 2.4 MTS assays

To assess the adherence of Ad-MSCs to scaffolds, the number of viable cells remaining at the bottom of each well after removal of bone discs was measured 1 day after seeding using an MTS assay (CellTiter 96^®^ AQueous One Solution Cell Proliferation Assay; Promega, Madison, WI, USA). Cell adherence rate was calculated with the following formula: (number of cells seeded − number of cells remaining on well bottom)/number of cells seeded. An MTS assay was also used to assess cell proliferation in each treatment group by measuring the number of viable cells. After 1, 2, and 3 days of culture, bone discs were washed with PBS and transferred to a fresh 96-well plate, before being incubated with 20% MTS reagent in serum-free medium at 37 °C in 5% CO_2_. After 2 h, aliquots were pipetted into a 96-well plate. Light absorbance by the contents of each well was measured at 490 nm using a spectrophotometric plate reader (SmartSpec 3000 Spectrophotometer; Bio-Rad, Hercules, CA, USA) [11]. MTS assay was repeated three times with five or more bones in each group.

### 2.5 Micro-computed tomography (micro-CT)

The ratio of demineralized to non-demineralized bone was measured non-destructively under water using a SkyScan 1172 micro-CT scanner (Micro Photonics, Allentown, PA, USA). A custom-prepared jig was used to position specimens. Micro-CT settings were as follows: image pixel size, 9.86 μm; exposure time, 590 ms; voltage, 70.0 kV; current, 141.0 μA; rotation range, 180°. Estimation of the proportion of demineralized bone and porosity for each experimental group were performed using CT-Analyser software (version 1.14.4.1; Bruker microCT, Kontich, Belgium). More than 900 sections of bone specimens were analyzed for each experimental group.

### 2.6 Scanning electron microscopy (SEM)

The surfaces of bone discs and distribution of Ad-MSCs were visualized by SEM (SUPRA 55VP; Carl Zeiss, Oberkochen, Germany) 3 days after cell seeding. No cells were observed on the non-seeded control discs. For observation of bone disc surfaces, samples were fixed in Karnovsky fixative (2% glutaraldehyde) at 4 °C for 2 h. Subsequently, samples were washed with 0.05 M sodium cacodylate buffer and post-fixed with 2% osmium tetroxide and 0.1 M cacodylate buffer for 2 h. Samples were then washed twice in distilled water and dehydrated using a series of ethanol solutions (30, 50, 70, 80, 90, 100, 100, and 100%) for 10 min at each step. Drying was carried out by two 10 min incubations in hexamethyldisilazane and placing discs in a drying oven overnight. After pre-treatment, samples were coated with platinum in an EM ACE200 vacuum coater (Leica Microsystems, Wetzlar, Germany) and mounted on stubs prior to observation by SEM. Three samples were photographed using the SEM for each group.

### 2.7 Real time polymerase chain reaction (RT-PCR)

For RT-PCR, Ad-MSCs were seeded on bone discs of each group and cultured for 3 days in 96-well plates. Total RNA was isolated from these cells using an RNA extraction kit (Hybrid-R™; GeneAll Biotechnology, Seoul, Korea). Complementary DNA (2 μl) was mixed with AMPIGENE™ qPCR Green Mix Hi-ROX (Enzo Life Sciences, Farmingdale, NY, USA) and forward and reverse primers, then amplified on an ABI StepOnePlus™ Real-Time PCR System (Applied Biosystems, Foster City, CA, USA). To assess the osteogenic potential of canine Ad-MSCs on bone discs, expression of the genes runt-related transcription factor 2 (*RUNX2*), alkaline phosphatase (*ALP*), *BMP-7*, and transforming growth factor β (*TGF-β*) was measured (Table 1). More than 10 samples were used for each group and all RT-PCR experiments were repeated 3 times.

**Table 1:** Primers used for RT-PCR.

Target	Sequence (5′–3′)
*GAPDH*	Forward CATTGCCCTCAATGACCACT
	Reverse TCCTTGGAGGCCATGTAGAC
*RUNX2*	Forward TGTCATGGCGGGTAACGAT
	Reverse TCCGGCCCACAAATCTCA
*ALP*	Forward TCCGAGATGGTGGAAATAGC
	Reverse GGGCCAGACCAAAGATAGAG
*BMP-7*	Forward TCGTGGAGCATGACAAAGAG
	Reverse GCTCCCGAATGTAGTCCTTG
*TGF-β*	Forward CTCAGTGCCCACTGTTCCTG
	Reverse TCCGTGGAGCTGAAGCAGTA

### 2.8 Statistical analysis

Data was expressed as means + SDs. Statistical analysis was performed using SPSS version 23.0 (SPSS Inc., Chicago, IL, USA). The Kruskal–Wallis test was used to analyze differences among groups, and the Mann–Whitney U test was carried out for post-hoc analysis. *P*-values < 0.05 were considered statistically significant.

## 3 Results

### 3.1 Demineralized bone morphology

**Fig. 2:**
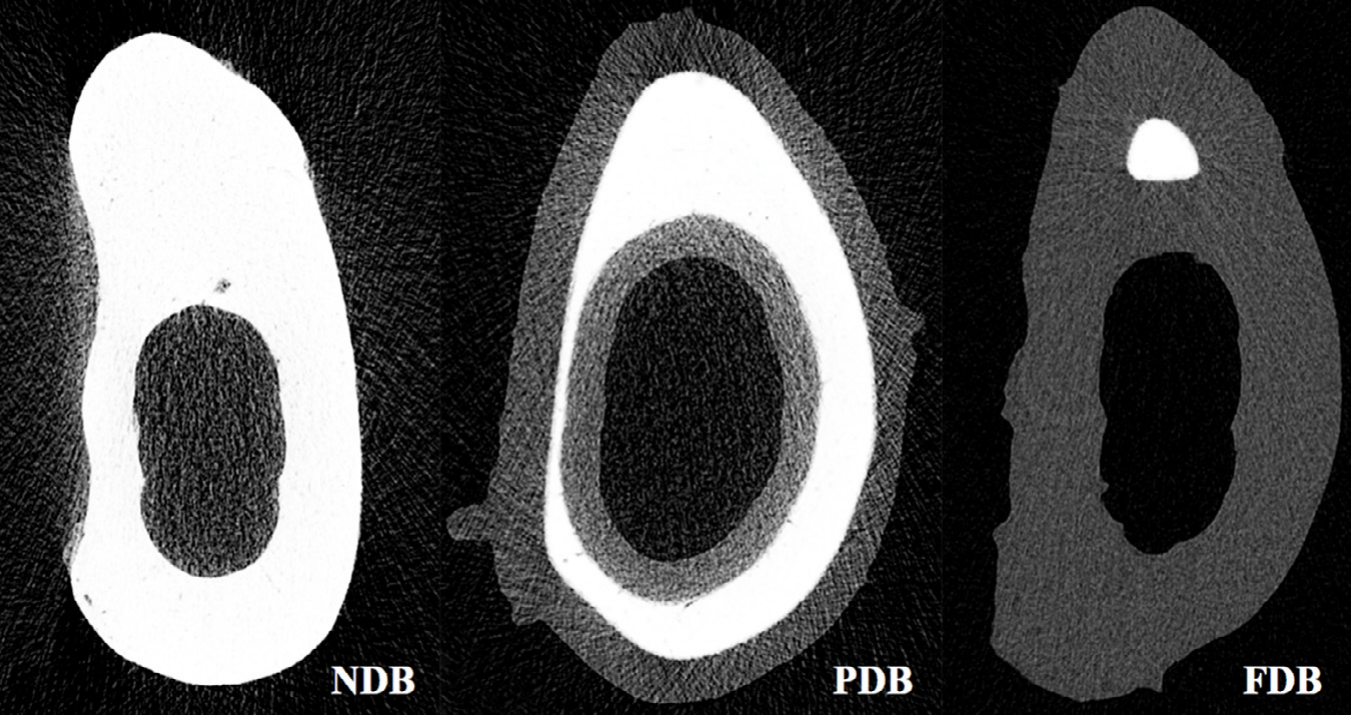
Micro-CT images showing bone demineralization in relation to HCl exposure time (increasing from the left panel to the right). White and grey regions represent non-demineralized and demineralized tissue, respectively. Demineralization and porosity rates were 0 and 1.22% for NDB, 57.13 and 20.52% for PDB, and 92.30 and 36.99% for FDB, respectively.

Micro-CT analysis revealed that the entire cortical bone was mineralized in NDB samples, appearing white in color (Fig. 2). Demineralization was seen to occur from the outer and inner surfaces. Demineralization rates of PDB and FDB samples were 51.3 and 92.3%, respectively. Porosity ratios in the NDB, PDB, and FDB groups were 1.22, 20.52, and 36.99, respectively (Table 2).

**Table 2:** Results of 3D analysis by micro-CT.

	NDB	PDB	FDB
Non-demineralization (%)	100	42.87	7.70
Demineralization (%)	0	57.13	92.30
Porosity (%)	1.22	20.52	36.99

**Fig. 3:**
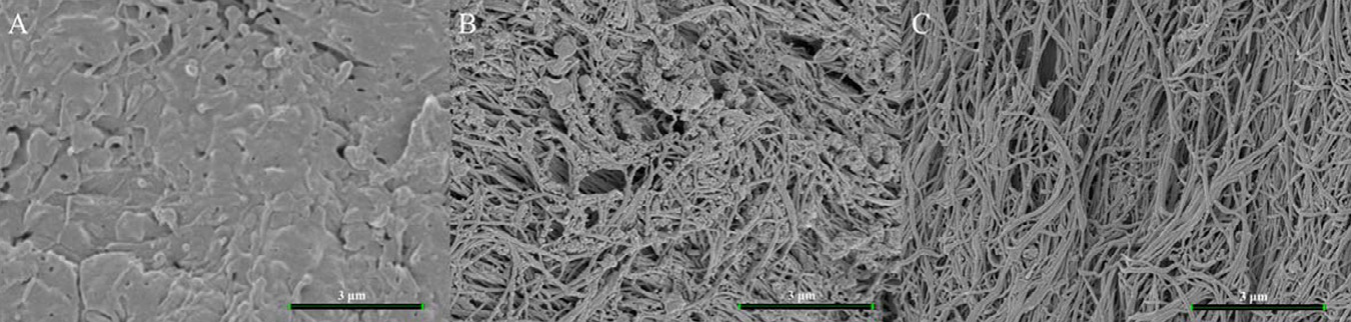
SEM images of bone disc surfaces at 30,000 × magnification. A. NDB; the disc surface was covered by mineral components. B. PDB; mineral components were partially removed and collagen fibers exposed. C. FDB; collagen fibers were completely exposed and free of mineral components.

SEM showed the surface of NDB samples to be irregular and almost completely covered by mineral components (Fig. 3), whereas collagen fibers were observed in PDB and FDB specimens. In particular, the surface of FDB exhibited numerous collagen fibers, appearing as twisted threads. Small mineral deposits were evident on the collagen fibers in PDB; however, FDB demonstrated no such mineralization.

### 3.2 Ad-MSC adherence to and proliferation on bone discs

**Fig. 4:**
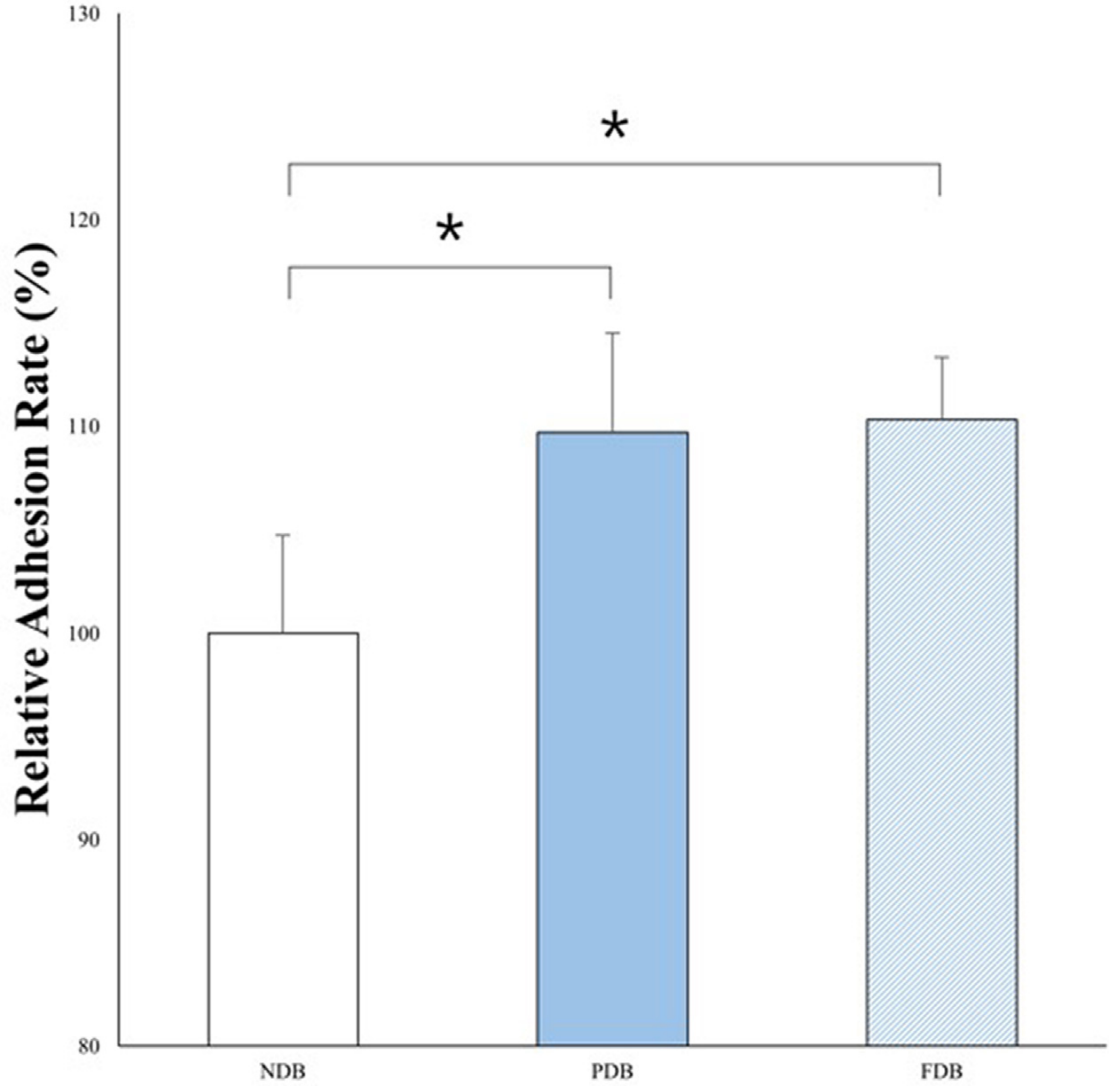
Adherence rates of Ad-MSCs to bone discs of each treatment group. Adherence of Ad-MSCs to PDB and FDB was greater than that to NDB; however, no significant difference was established between PDB and FDB in this respect. *Indicates a statistically significant difference (*P* < 0.05) between groups. Each bar represents the mean ± SD of 6 samples.

**Fig. 5:**
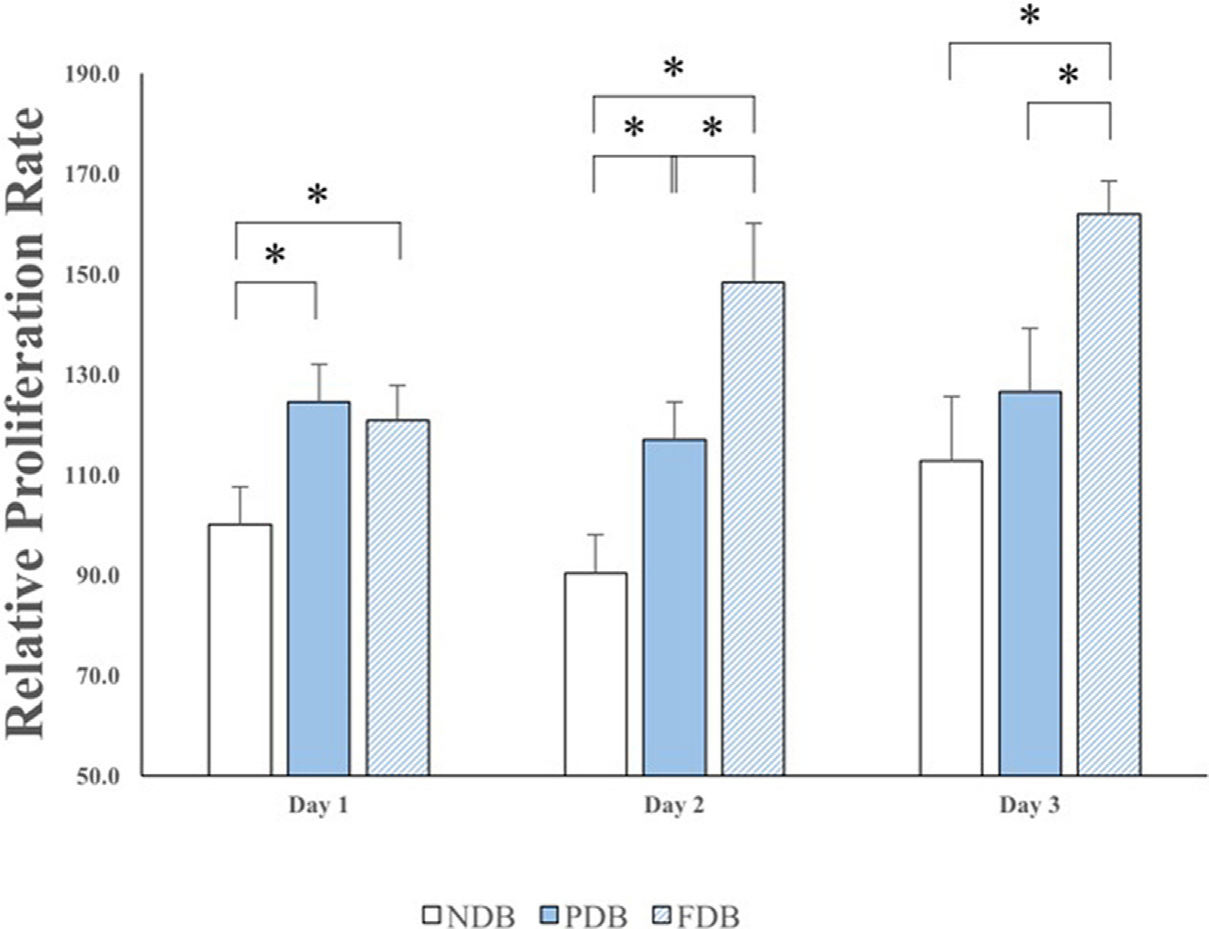
Ad-MSC proliferation rates on bone discs at days 1, 2, and 3 of culture. Day 1: Ad-MSC proliferation rates in the PDB and FDB groups were higher than that in the NDB group. Day 2: Only FDB exhibited increased Ad-MSC proliferation rates. Day 3: Proliferation on FDB was seen to have gradually increased from day 1, in contrast to the other treatment groups. *Indicates a statistically significant difference (*P* < 0.05) between groups. Each bar represents the mean ± SD of 6 samples.

**Fig. 6:**
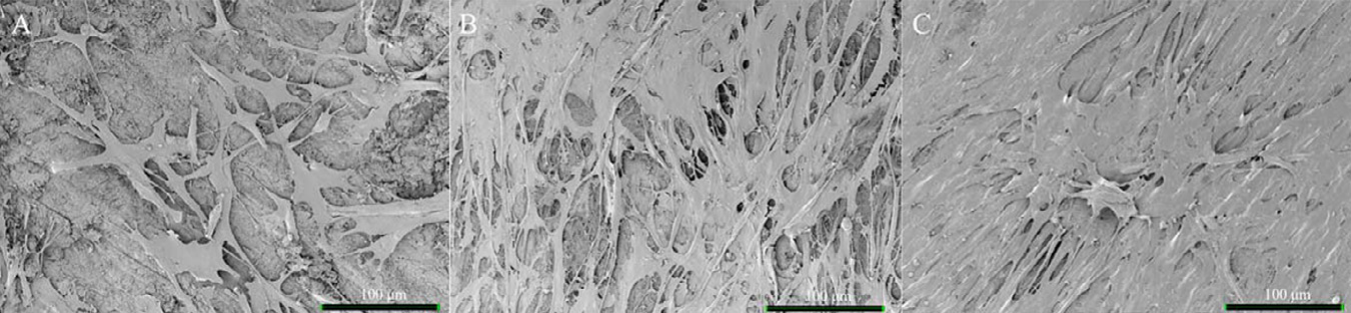
Ad-MSCs cultured for 3 days on bone discs in low-glucose DMEM at 800 × magnification. A. NDB; B. PDB; C. FDB; On NDB, only small fraction of the surface was covered with Ad-MSCs. Denser on PDB and the entire surface was covered with Ad-MSCs on FDB.

Relative adherence rates in the NDB, PDB, and FDB groups were 100, 109.71, and 110.33%, respectively (Fig. 4). The difference between PDB and FDB samples in this respect was not significant, although adherence rates in both of these groups were significantly higher than that in the NDB group (*P* < 0.05; Fig. 4). Proliferation rates in the FDB group increased gradually over time, but those in the other treatment groups did not (Fig. 5). From day 2 after cell seeding, proliferation was significantly higher using FDB than NDB or PDB (*P* < 0.05). Ad-MSCs were able to attach to and grow on all three bone disc types; however, those cultured on FDB appeared in a stacked formation (Fig. 6).

### 3.3 Osteogenic gene expression in Ad-MSCs

**Fig. 7:**
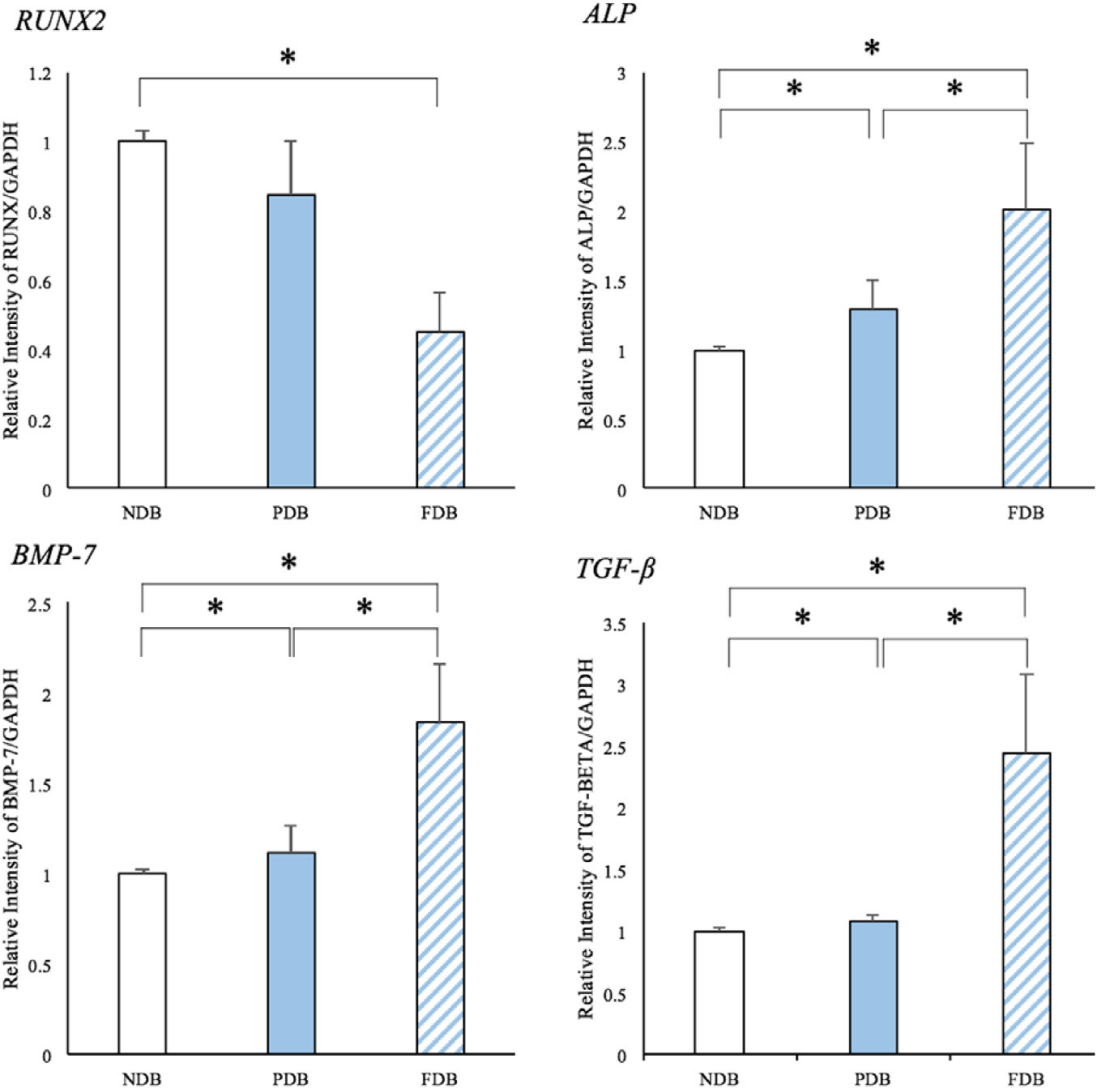
Expression of *RUNX2*, *ALP*, *BMP-7*, and *TGF-β* genes 3 days after cell seeding. *Indicates a statistically significant difference (*P* < 0.05) between groups. Each bar represents the mean ± SD of 6 samples.

*RUNX2* expression of Ad-MSCs on FDB was significantly lower than that of those cultured on NDB (*P* < 0.05; Fig. 7). However, *ALP*, *BMP-7*, and *TGF-β* transcription by Ad-MSCs of the FDB group was significantly elevated compared to the other groups. Specifically, expression levels of these osteogenic genes in Ad-MSCs on FDB were more than twice those observed in the NDB group.

## 4 Discussion

In the present study, SEM and micro-CT showed that HCl demineralization for 48 h successfully removed the mineral components of bone, exposing the organic constituents. In addition, demineralization increased bone porosity. Demineralization triggers changes in the mechanical and biological properties of bone [12]. Higher porosity results in a larger surface area, contributing to osteogenic protein adsorption, as well as ion exchange [13]. Moreover, cell attachment, proliferation, and differentiation may be enhanced by increased surface roughness related to bone porosity [13].

Here, bone disc demineralization was found to enhance Ad-MSC adherence more than non-demineralization of it. Organic bone components, such as type I collagen, are exposed following demineralization [14]. These demonstrate chemotactic or adhesive characteristics, as they contain arginine-glycine-aspartic acid (RGD) peptides that mediate cellular adhesion [15]. The greater the degree of type I collagen exposure by demineralization, the more cell adhesion is promoted by the RGD sequence. Thus, the different adhesion rates observed among NDB, PDB, and FDB may be explained by varying accessibility of the RGD peptide. In the current work, Ad-MSCs on FDB exhibited higher proliferation rates as well. Differences in proliferation between treatment groups might also be explained by the RGD sequence. As RGD-mediated cell attachment occurs, transmembrane receptors trigger signaling cascades that initiate cell proliferation [16].

Three days after cell seeding, we found expression of *BMP-7*, *ALP*, and *TGF-β* in the PDB and FDB groups to be upregulated compared with the NDB control. However, *RUNX2* transcript levels in the former two groups were downregulated compared with the latter. Bone contains osteogenic proteins, such as those of the BMP subfamily and *TGF-β* [17]. BMPs are exposed by demineralization and might contribute to Ad-MSC osteogenic differentiation through SMAD and MAPK pathway [18]. *RUNX2* is a transcription factor required for bone formation and a major target of *TGF-β* and BMP signaling [19]. BMP subfamily proteins can upregulate *RUNX2* [19]; however, such elevated expression is only transiently maintained and begins to decrease soon after the initiation of upregulation [19]. Therefore, the observed downregulation of *RUNX2* may be the eventual consequence of promoted expression by members of the BMP subfamily.

Based on the above results, demineralization may enhance the biocompatibility of bone allografts for use in bone regeneration. Fully demineralized cortical bone might be a suitable allograft material capable of stimulating the osteogenic differentiation of Ad-MSCs.

## Declarations

### Author contribution statement

Kwangrae Jo: Performed the experiments; Analyzed and interpreted the data; Contributed reagents, materials, analysis tools or data; Wrote the paper.

Yongsun Kim, Wan Hee Kim, Oh-Kyeong Kweon: Conceived and designed the experiments.

Seung Hoon Lee, Yong Seok Yoon: Contributed reagents, materials, analysis tools or data.

### Funding statement

This work was supported by the National Research Foundation of Korea (2015R1D1A1A-01057415).

### Competing interest statement

The authors declare no conflict of interest.

### Additional information

No additional information is available for this paper.
